# Cable bacteria with electric connection to oxygen attract flocks of diverse bacteria

**DOI:** 10.1038/s41467-023-37272-8

**Published:** 2023-03-23

**Authors:** Jesper J. Bjerg, Jamie J. M. Lustermans, Ian P. G. Marshall, Anna J. Mueller, Signe Brokjær, Casper A. Thorup, Paula Tataru, Markus Schmid, Michael Wagner, Lars Peter Nielsen, Andreas Schramm

**Affiliations:** 1 Center for Electromicrobiology (CEM), Section for Microbiology, Department of Biology, Aarhus University, Aarhus C, Denmark; 2 Microbial Systems Technology Excellence Centre, University of Antwerp, Wilrijk, Belgium; 3 Centre for Microbiology and Environmental Systems Science, Division of Microbial Ecology (DOME), University of Vienna, Vienna, Austria; 4 Doctoral School in Microbiology and Environmental Science, University of Vienna, Vienna, Austria; 5 Bioinformatics Research Center (BiRC), Aarhus University, Aarhus C, Denmark; 6 Center for Microbial Communities, Department of Chemistry and Bioscience, Aalborg University, Aalborg, Denmark

**Keywords:** Water microbiology, Cellular microbiology, Microbial ecology

## Abstract

Cable bacteria are centimeter-long filamentous bacteria that conduct electrons via internal wires, thus coupling sulfide oxidation in deeper, anoxic sediment with oxygen reduction in surface sediment. This activity induces geochemical changes in the sediment, and other bacterial groups appear to benefit from the electrical connection to oxygen. Here, we report that diverse bacteria swim in a tight flock around the anoxic part of oxygen-respiring cable bacteria and disperse immediately when the connection to oxygen is disrupted (by cutting the cable bacteria with a laser). Raman microscopy shows that flocking bacteria are more oxidized when closer to the cable bacteria, but physical contact seems to be rare and brief, which suggests potential transfer of electrons via unidentified soluble intermediates. Metagenomic analysis indicates that most of the flocking bacteria appear to be aerobes, including organotrophs, sulfide oxidizers, and possibly iron oxidizers, which might transfer electrons to cable bacteria for respiration. The association and close interaction with such diverse partners might explain how oxygen via cable bacteria can affect microbial communities and processes far into anoxic environments.

## Abstract (web summary)

Cable bacteria are centimeter-long filamentous microbes that conduct electrons via internal wires, thus coupling sulfide oxidation between sediment layers. Here, Bjerg et al. show that the anoxic part of oxygen-respiring cable bacteria attracts swarms of other bacteria, which appear to transfer electrons to cable bacteria via soluble metabolites.

## Introduction

Cable bacteria are long filamentous bacteria that can transmit electrons over centimeter distances and thereby couple the oxidation of sulfide to the remote reduction of oxygen or nitrate^1,2^. They occur globally in marine and freshwater sediments and aquifers^3–5^ and interfere directly with sulfur, oxygen, carbon, and nitrogen cycling^6^. Via pH gradients and electric fields induced by their relocation of electrons, they also indirectly influence iron, calcium, cobalt, and arsenic cycling and all ion fluxes in their habitats^6–9^. In freshwater sediments, they can cause a 4.5-fold stimulation of sulfate reduction and drastically lower methane emission^10,11^.

Cable bacteria activity has also been linked to enhanced carbon assimilation of autotrophic sulfide oxidizers^12^ and correlated with the distribution of iron-cycling bacteria in marine sediments^13^. These apparent associations have led to speculations that bacteria in anoxic sediment somehow may use cable bacteria as an electron conduit to oxygen^14,15^.

Here we use a combination of microscopic observations, metagenome sequencing, laser microdissection, and Raman microscopy to demonstrate the dynamics and intimacy of this association, tentatively identify the bacteria involved, and propose a likely mechanism for electron transfer between associated bacterial flocks and cable bacterial filaments.

## Results and discussion

### Bacteria flock around cable bacteria and appear metabolically stimulated

**Fig. 1:**
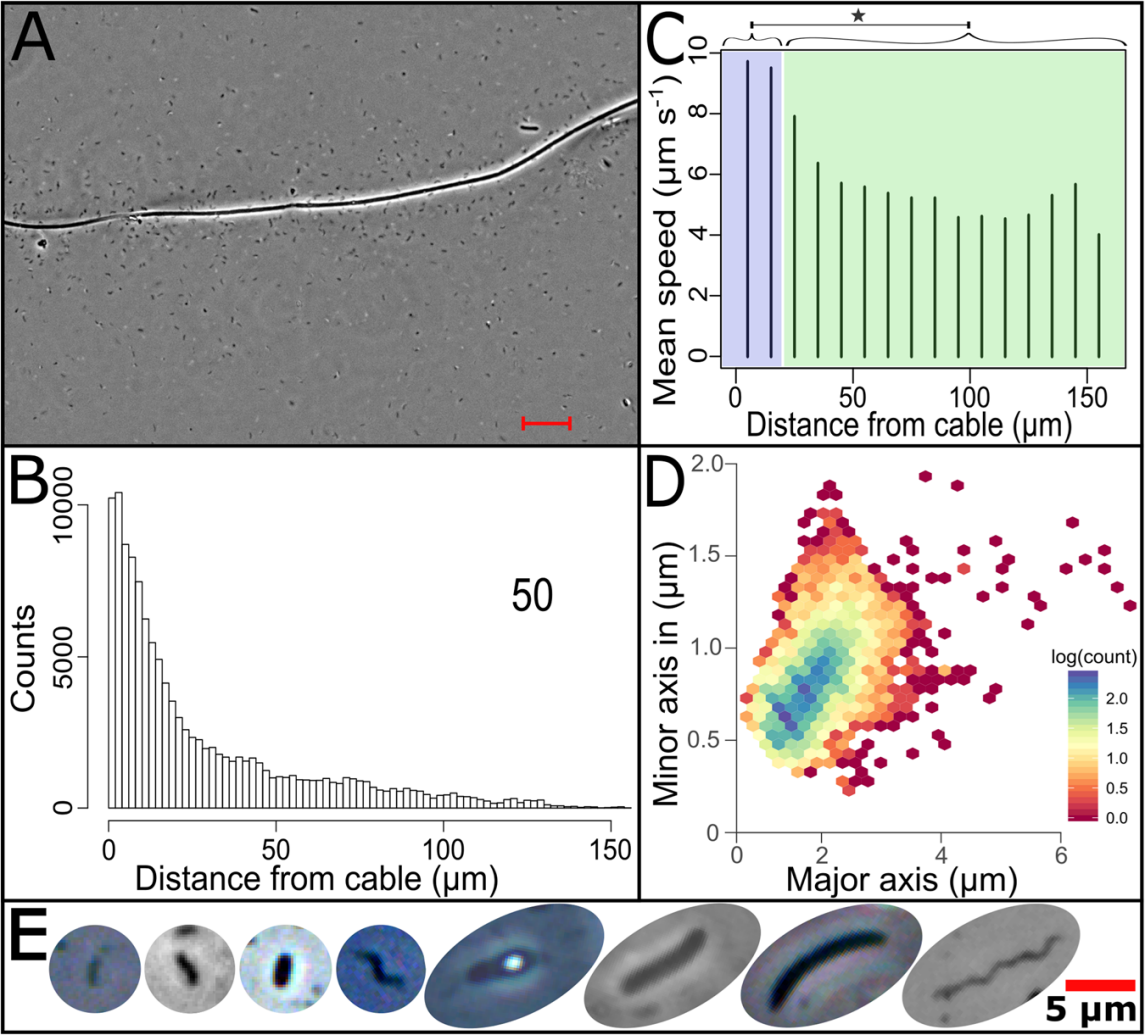
Documentation and main properties of bacterial flocks around cable bacteria. **A** Flocking bacteria attracted to a cable bacterium filament (center) (Scale bar, 10 µm). **B** Counts of swimming bacteria at different distances to the cable bacterium filament (960,071 counts of 3211 flocking bacteria in 12 video frames, mean distance 17.42 µm. The means of the individual samples ranged from 4.96 to 24.58 µm. **C** Difference in mean swimming speed of bacterial cells relative to their distance to the cable bacterium filament. The shaded blue area corresponds to a distance within 20 µm of a cable bacterium, shaded green to more than 20 µm. Welch’s two-sample *t*-test (two-sided) shows that the swimming speed of cells is significantly different between these two distance groups (indicated by *); *p*-value = 2.2e^−16^ (*N*_samples_ = 11, *N*_cells_ = 2712). **D** Density plot of bacterial cell sizes from all samples (*n* = 12), showing that the majority of interacting cells is small. **E** Phase contrast images of the different cell morphologies found. Source data are provided as a Source Data file.

Cable bacteria from an enrichment of the freshwater strain *Ca*. Electronema aureum GS^16^ were observed under semi-natural conditions on a microscope slide (a so-called trench slide), where oxygen diffused in from the edge of the coverslip, while organic matter, sulfide, and other nutrients were provided from sediment in a central compartment (trench)^17,18^. This setup established an observation zone where cable bacteria stretched out from the sediment to the oxic–anoxic interface, which was clearly delineated by a microaerophilic veil of motile, aerobic bacteria (Fig. S1). In the anoxic part of the zone, up to 4 mm away from the oxic-anoxic interface, bacterial cells were discovered to swim in a flock around cable bacteria segments, generally outnumbering adjacent cable bacteria cells by 2.2:1 (Fig. 1A, Movie S1, Table S1). Detailed cell tracking showed chemotactic behavior towards the cable bacteria: the flocking cells were concentrated close to the cable bacteria filaments, with the highest cell densities within a distance of 20 µm but still increased densities until at least 50 µm away (Fig. 1B, Fig. S2). There was no overt pattern of the flocking bacteria touching cable bacteria, but with the resolution limitations of conventional light microscopes and the dynamic nature of the interaction deterring higher resolution methods, we currently cannot exclude that touching might occur; but if so, it was rare and brief. The swimming velocity of the flocking bacteria was significantly enhanced within a distance of 20 µm (Fig. 1C), indicating an increased proton motive force^19–21^. The fraction of bacterial cells detectable by fluorescence in situ hybridization (FISH) within 10 µm distance from cable bacteria was significantly higher than >10 µm away (Table S2), suggesting that they had a higher ribosome count and thus higher metabolic activity compared to bulk sediment bacteria^22^. Taken together, this indicates high metabolic rates within the flock and metabolic stimulation of the flocking bacteria when getting really close to the cable bacteria.

### Bacterial flocks disperse when cable bacteria are disconnected from oxygen

**Fig. 2:**
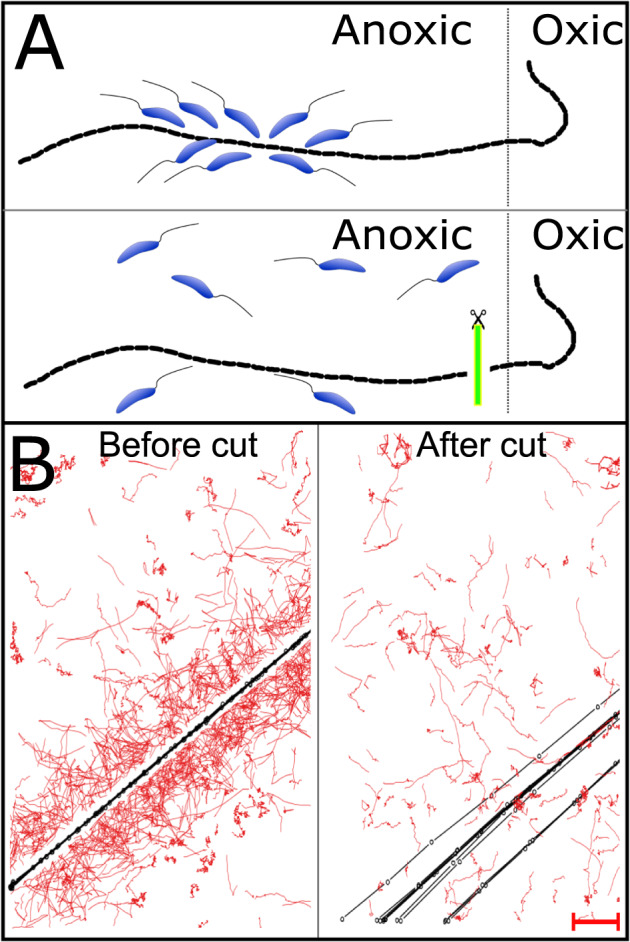
Principle and example of results of the laser cut experiment (*n* = 9). **A** Schematic representation. A cable bacterium connected to oxygen at the right side is cut near the oxic-anoxic interface using a laser microdissection microscope. **B** Result. Bacterial tracks on the suboxic part of a cable bacterium generated from a video before and after a cut. Red lines are tracks of swimming bacteria, and black lines and dots denote the position of the cable bacterium for every 50 frames. The cable bacterium moves more after the cut as if the filament begins its oxygen chemotaxis (Scale bar, 10 µm). Source data are provided as a Source Data file.

Flocks of swimming bacteria were commonly observed in our setup, i.e., on 17 of 21 cable bacteria in contact with oxygen; in contrast, flocks were never observed on cable bacteria not in contact with oxygen, supporting a dependency of the chemotaxis on a high-potential electron sink in the cable bacteria^18^. This was strikingly confirmed by the swift response of the bacterial flocks upon cutting the cable filament in two with a laser (Fig. 2): flocking around the part now cut off from its electrical connection to oxygen ceased within 13 ± 2 s (Table S3). When a cable bacterium filament was cut in the middle of a bacterial flock, the response was even more evident. The flocking bacteria immediately dispersed from the part of the filament that was no longer connected to oxygen but was still observed around the part that still retained its connection to oxygen (Movie S2). This instant response suggests that the flocking bacteria are attracted by a condition created by cable bacteria exclusively while they conduct electrons to oxygen, and not, for example, extracellular polymers excreted as part of their motility^17^.

### Cable bacteria associates are morphologically, phylogenetically, and metabolically diverse

**Fig. 3:**
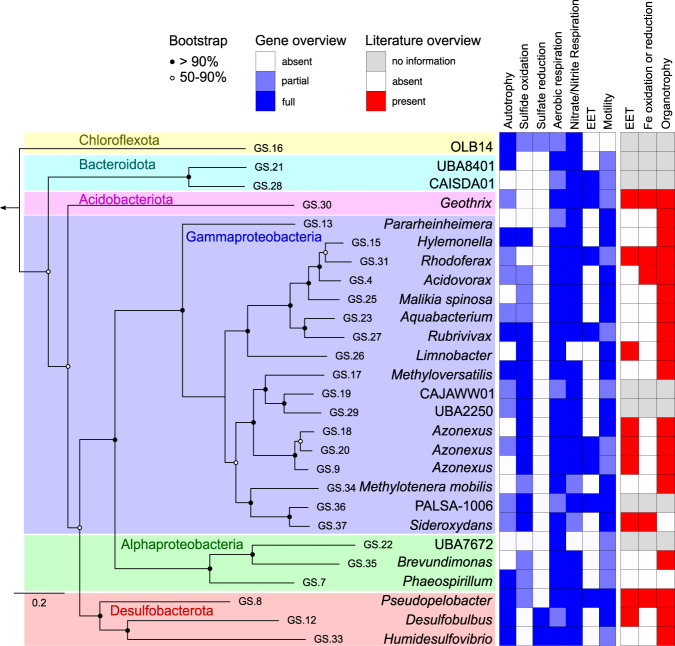
Genome-based phylogeny and selected key features of putative cable bacteria-associated bacteria. Data were derived from the annotation of metagenome-assembled genomes and from literature searches. EET, extracellular electron transfer. Source data are provided in Supplementary Datasets 1–3.

The bacteria interacting with the freshwater cable bacteria were morphologically highly diverse. The majority were rod-shaped, vibrioid, or ovoid cells of 1–2 µm size (long axis), but a few distinct larger cell types, including straight rods, curved rods, and spirochete-like cells, were also recorded (Fig. 1D, E, Fig. S3). To get insights into the likely identity and metabolic diversity of the bacteria forming the flocks, we assembled 27 good-quality genomes (Fig. 3, Supplementary Datasets 1–3) from a metagenome prepared by sampling the cable bacteria-observation zone of one of the microscopy slides (Fig. S1). We identified 25 genera from 6 phyla, which, based on genome annotation and comparison to their closest relatives, had four major unifying traits: motility, chemotaxis, organotrophy, and respiration (with oxygen/nitrate/nitrite) (Fig. 3). In addition, sulfide oxidation and autotrophy were common, while sulfate or iron reduction and iron oxidation were rare; methane oxidation was never identified. These data are consistent with a scenario in which the flocking bacteria can oxidize organic compounds, sulfide, and possibly Fe^2+^ by delivering electrons to cable bacteria, i.e., they breathe via cable bacteria. While some of the extracted genomes do show genes for extracellular electron transfer (EET), and some of the closest relatives of those bacteria also exhibit EET capabilities (Fig. 3), there is no clear and consistent pattern for a known EET mechanism.

### Interspecies electron transfer via soluble intermediates likely explains the flocking

**Fig. 4:**
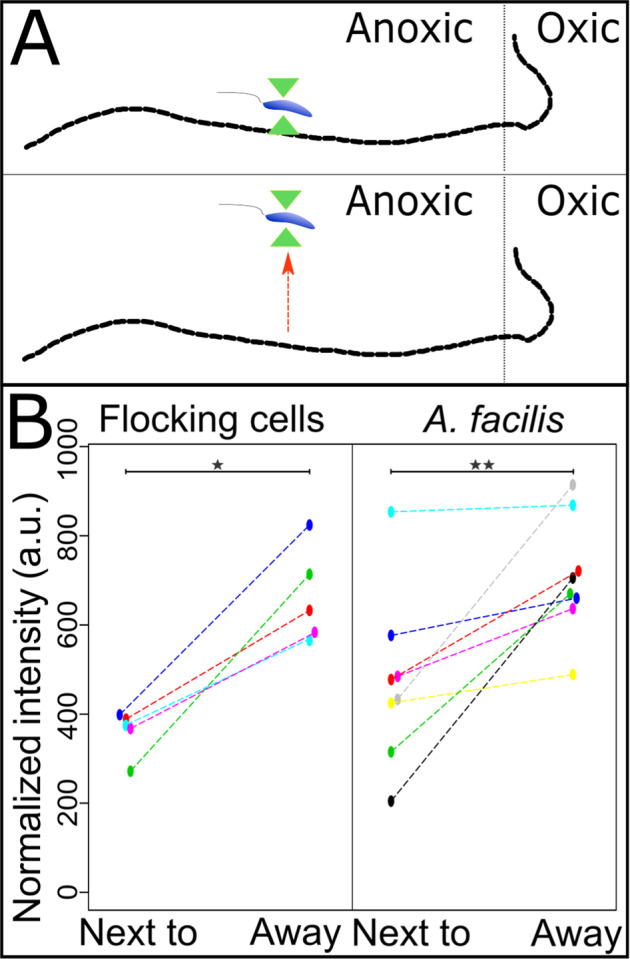
Cytochromes in flocking bacteria are more oxidized when close to a cable bacterium. **A** Schematic representation of the experiment. A flocking cell is captured and moved right next to a cable bacterium filament, measured by Raman microscopy, then moved ~50–100 µm away and measured again after ~3 s. **B** The change in normalized intensity of the 750 cm^−1^ band for each individual cell when moved next to and away from the cable bacterium. The 750 cm^−1^ band is indicative of cytochrome redox state, with high values for reduced and low values for oxidized cytochromes. Band intensities and, thus, cytochrome redox states are significantly different between the two positions (*N*_native cells_ = 5, *p*-value = 0.015, indicated by *; *N*_*A.facilis*_ = 8, *p*-value = 0.0387 indicated by **, two-sided *t*-test for dependent samples,); a.u., arbitrary units. Source data are provided as a Source Data file.

Electrons can be exchanged between prokaryotic cells by direct contact, by transfer through protein nanowires or conductive materials, or mediated through soluble electron shuttles^23^. The apparent chemotactic behavior with constant swimming and no stop-and-touch behavior, as observed in some electron transfers to minerals^24^, suggests that interspecies electron transfer (IET) from flocking bacteria to cable bacteria is mediated through gradients of soluble intermediates. This was further supported by observations of redox states of cellular cytochromes with Raman microscopy^18^; cells of the flocking bacteria captured and moved with a laser tweezer were significantly more oxidized when placed closer than 5 µm to the cable bacterium and more reduced when more than 50 µm away (Fig. 4, Fig. S4). The same significant change was observed with cells introduced into the slides from a culture of *Acidovorax facilis* DSM649, a cultured representative of one of the most abundant members of the associated bacterial community (Fig. 3; 96.03% average nucleotide identity (ANI) to genome bin GS.4 recovered in this study); cytochrome redox state did not change in control cells randomly moved with the laser tweezer (Fig. S5).

We estimate that shuttle concentrations in the nM range are sufficient to support the respiration of the bacterial flock (Supplementary Note 1, Table S4). Since aquatic sediments can contain high-potential shuttle compounds exceeding this concentration (e.g. >1 mg ml^−1^ humic acids in lake sediment^25^, or flavins in the nM range in marine sediment^26^), shuttles do not have to be produced by cable bacteria or associated bacteria. Also, the turnover rate of a soluble mediator in this concentration range would be below 2 s (Supplementary Note 1, Table S4). This rapid turnover suggests an immediate depletion of the oxidized mediator once cable bacteria stop re-oxidizing its reduced form and thus explains the rapid dispersal of the bacterial flock upon cutting the connection of the filament to oxygen. That cable bacteria provide a potent oxidized electron shuttle seems the only plausible explanation for the proliferation of other bacteria associated with cable bacteria in the anoxic sediment compartment^12,13^, the amount and vigor of motile cells surrounding cable bacteria (Fig. 1A, Movie S1), and their rapid dispersal after cutting (Movie S2).

On the cable bacteria side, no known EET capabilities have been identified in cable bacteria genomes^27^, yet intact cable bacteria have successfully been connected to electrodes^28^, making it likely that an undescribed outer membrane electron conduit exists, which is able to receive electrons from a shuttle^28^.

### Conclusion and perspectives

Cable bacteria attracting and intimately engaging with a diversity of other bacteria in sediment is yet another surprise finding in relation to the electrical currents first derived from geochemical anomalies and later ascribed not to conductive minerals or nanowires but living wires in the form of the centimeter-long cable bacteria^1,29^. Now a line of new and interesting questions for future research arises: How widespread and important are these interactions in situ? What specific molecules mediate the interactions, and how and where are they processed in the cells on either side? How much of the current in a cable bacterium does arise from the bacterial flocks around it, and what share may the flock gain of the energy conserved from the primary oxidation processes to the final oxygen reduction? Is the interaction beneficial or detrimental for the cable bacterium, and is it controlled in any way? Are there harder-to-observe interactions with other, non-motile cells in the natural sediment environment, and what is the full palette of microbial processes stimulated by the electrical shortcut to oxygen offered by cable bacteria?

## Methods

### Sampling and incubation

All experiments were conducted with a freshwater sediment enrichment culture of *Ca*. Electronema aureum GS, containing a single cable bacterial species and many native and cable bacteria-associated freshwater sediment bacteria^16^. This enrichment was established by heat-treating pond sediment collected at the Aarhus University campus in Denmark and inoculating it with a single cable bacterium filament. The resulting clonal cable bacterium enrichment has been maintained and transferred for >7 years in our laboratory.

### Glass slide chambers and microscopy

Custom-made glass slide chambers^17^ were constructed for the microscope video, manipulation experiments, and Raman microscopy: glass slabs were glued onto a microscopy slide, creating a trench in the middle, which was filled with sediment from the *Ca*. Electronema aureum GS enrichment culture and covered with N_2_-flushed tap water and a cover slip, creating a 60 × 22 mm space, of which 40 × 12 mm was taken up by the trench, with a separation between coverslip and slide of roughly 150–200 µm (Fig. S1).

Slides were incubated in a moist environment for 2–24 h and then investigated on an AxioObserver microscope (Zeiss, Germany) equipped with a motorized stage.

Most recordings and observations were made at 100× magnification with dark field illumination, but for tracking and distribution data, high-frame-rate videos were recorded at 400× magnification in phase contrast, with images taken 88 or 38 ms apart. Only videos of sufficient quality and length, with a single cable bacterium present and without major sediment particles, were used for subsequent analysis.

### Cell tracking

To quantify cell movement (swimming) within the bacterial flocks around cable bacteria, the recorded videos were analyzed in Fiji^30^. To remove background and non-moving objects, the median pixel value for the whole stack of images was subtracted, leaving only outliers, i.e., moving objects, and this was then further processed by using ‘enhance contrast’ and ‘sharpen’ before converting to a binary image. The threshold for this conversion and values for ‘enhance contrast’ were adjusted for each video to mark up the cable bacterium and cells without creating artifacts larger than 2 µm around the cable. To enable an analysis of the motile cells relative to the cable bacterium filament, the cable bacterium filament’s position was marked manually every 50th frame by measuring along it with Fiji’s ‘measure segmented line’ tool. After this, the Fiji plugin, Mtrack2, was used to count and record the position of every cell in the flock in each frame of each video. Manual tracks were made with the Fiji plugin MtrackJ^31^ of a small subset of cells. Large flocking bacteria and spirochete-type cells had to be manually tracked, as the constant shape change and into/out of focus movement made automatic tracking unreliable. Videos were analyzed with the goal of collecting data for the distance of each flocking cell to the cable bacterium filament, its size, and speed, and finally, tracking its movements. Settings were deliberately set wide in Mtrack2, as there were many different sizes and velocities of flocking cells (Fig. 1, Fig. S3). To get a count of cells, and an estimate of their morphological diversity, cell counts were done manually for a few frames, then Fiji’s ‘analyze particles’ tool was used to identify cell-sized objects between 0.4 and 12 µm diameter and a circularity of 0.1–0.8. These counts probably underrepresent the number of cells near the cable bacterium, as cells and tracks were lost when they were on or close to the cable bacterium due to the filament’s shadow and the light artifact it generates under phase contrast imaging. Frames with rapid movements of the cable bacterium filament^17^ or parts of the cable bacterium out of focus were excluded from the final analysis.

### Tracking and size analysis in R

All tracking and size results from the flocking bacteria and cable bacteria were saved in csv format and then subjected to further filtering and analysis in R. A custom script was used to filter the tracks. To remove artifacts where the tracking had incorrectly jumped from one cell to another, tracks were broken up when cells suddenly changed their average speed. Particles and cells being jostled by Brownian motion would generate tracks in Mtrack2, as would cells stuck to the glass surface. To remove these invalid tracks, any track which did not span more than 50 pixels on the *x*- or *y*-axis was removed, and only tracks which spanned longer than 10 s were kept for further analysis. Data on the behavior of the tracked cells were generated using a custom R script, which calculated the speed of a tracked cell, its traveled distance, and its distance to the nearest cable bacterium.

To make sure the analysis of cell morphology only used flocking cells attracted to and swimming around cable bacteria, the coordinates of each track were compared to the size data, and all size measurements for that track were reported. The mean of the minor and major axis of these cells was calculated. Tracks where size differed more than 20%, were removed to avoid artifact. A plot of tracked cells was manually inspected, and any obvious artifacts, like shadows from cable bacteria or out-of-focus elements, were removed prior to further analysis.

### Fluorescence in situ hybridization

Glass slide chambers with cable bacteria and bacterial flocks were incubated for 3 days, then dried at 46 °C for 90 min, after which the cover glass was removed. The sample was dehydrated by gently flooding with a graded ethanol series (50%, 75%, 96% ethanol, 3 min each). A circle was then drawn with a PAP-pen (Rockland Immunochemicals Inc. Limerick, PA, USA) around the area of interest to enclose the solutions during FISH. Hybridization with Cy3-labeled probes EUB338 I-III (5′- GCWGCCWCCCGTAGGWGT-3′; ordered from biomers.net), targeting almost all bacteria, was performed at 46 °C for 90 min using standard protocols^32^. The hybridization buffer contained 0.9 M NaCl, 20 mM Tris-HCl, 35% formamide, and 0.01% SDS. The washing step was modified as follows to avoid disturbance of the sample: the slide was placed on a pre-heated plate (50 °C), hybridization buffer was quickly removed by pipetting and paper dabbing, the slide was then covered with pre-heated washing buffer (80 mM NaCl, 20 mM Tris-HCl, 5 mM EDTA, 0.01% SDS), which again was removed by pipetting and paper dabbing; this washing procedure was repeated twice. Finally, the slide was air-dried, covered with 1 µg ml^−1^ of 4′,6-diamidino-2-phenyl indole (DAPI) in Vectashield-Citifluor (4:1) and analyzed on an epifluorescence microscope (Axiovert 200 M, Zeiss). A conservative threshold of 10 µm distance to the cable bacterium filament was used as a boundary for scoring members of the bacterial flocks to avoid including cells that might have been moved around as an effect of the washing steps.

### Laser dissection manipulation

Laser cutting of cable bacteria surrounded by flocks of swimming bacteria was performed on a Laser Microdissection microscope (LMD 7000, Leica, Germany). The bacterial flocks could be observed visually at 400× magnification but were not easily discernible in the camera system of the LMD. Therefore, imaging was performed by switching between the LMD and an AxioObserver (Zeiss) microscope with phase contrast. However, since the dispersal of flocks after cutting the cable bacterium was a rapid event, dispersal time was recorded with a stopwatch while looking through the ocular. Dispersal time was defined as the time span until no motile bacteria were observed within 15 µm of the filament, which corresponded to the observable area around the cable bacterium filament in the LMD. Only cable bacteria that could be traced from the oxic–anoxic veil to the area of the flock without becoming entangled in other filaments were used. For video recording, the position of the flocking bacteria was detected on the AxioObserver microscope and indicated on the back of the slide with a marker. The slide was then transferred to the LMD, the filament was cut with the laser, and the slide was immediately transferred back to the AxioObserver microscope. Videos were recorded for 1 min before and after the cut. The average transfer time, including finding the position of flocking bacteria again, was <1 min.

### Metagenome sequencing, genome assembly, and bioinformatics analysis

A metagenome was reconstructed from the pooled reads of five metagenomics sequence libraries described in Kjeldsen et al.^27^. The metagenome was binned using MetaBat version 0.25.4 with the parameter –superspecific^33^. Coverage files for differential coverage analysis were generated by mapping the reads from the five sequence libraries onto the metagenome using BBMap version 35.82 with a minimum sequence identity threshold of 98% and converted to the bam file format using SAMtools version 1.9^34,35^.

To identify potential cable bacteria-associated microbes, the reads from a sequence library originating from a glass slide chamber were used. As described in Kjeldsen et al.^27^, this library was obtained with a sediment enrichment of *Ca*. Electronema aureum GS added to a glass slide chamber (Fig. S1). The cable bacteria and associated bacteria that had moved out of the sediment towards the oxic-anoxic interface and established in the transparent zone were flash-frozen on a slab of aluminum cooled with dry ice and then scraped off with a sterile razor blade after lifting off the coverslip (Fig. S1). DNA was extracted using the PowerLyser® DNA Isolation Kit (MoBio Inc.). Libraries for metagenomic sequencing of the extracted DNA were prepared using the Nextera DNA Library Preparation Kit (Illumina) and sequenced using the Illumina MiSeq kit v3. Reads were quality-checked using FastQC^36^ version 0.11.4 and trimmed using Trimmomatic v. 0.33^37^. Reads from this sequencing library were mapped back to the bins using BBMap^34^. Bins with more than 70% of their scaffolds covered by reads from this library were considered cable bacteria associates. Completeness and contamination of these genome bins were assessed using CheckM version 1.1.3 with the lineage workflow set to “Bacteria”^38^. Genomes more than 70% complete and with less than 5% contamination were used for further analysis. These genomes were classified using GTDB-tk version 1.5.1 and release 202 of the GTDB database^39^. The concatenated alignment from GTDB-tk was used to generate a phylogenetic tree using IQ TREE version 1.6.12^40^ with 100 bootstraps, and the model parameter *m* was set to TESTNEW.

Genome bins were functionally characterized by kofamscan version 1.2.0 and by blastp in BLAST version 2.11.0+ against a database of protein sequences related to extracellular electron transfer, specifically OmcS, MtrC, MtrF, OmcA, PioA and PilA (see Supplementary Dataset 1 for accession numbers)^41,42^. Kofamscan hits were considered matches when the lowest e-value matched the query model and was below 1E−6. Modules or categories where fewer than 10% of proteins matched were considered absent, 10–70% considered partially present, and 70–100% considered fully present. BLAST matches were considered hits with >30% sequence identity over 70% of the length of the query sequence. A single positive BLAST match for an EET-related gene (see Supplementary Dataset 1) was considered a full match. Dissimilatory sulfite reductase (DsrAB) proteins were determined as being oxidative or reductive based on the best BLAST hit against a DsrAB database^43^. These combined kofamscan and BLAST methods were used to determine the traits shown in Fig. 3. Sulfide oxidation determination was based on the presence/absence of genes encoding reverse DsrAB or the Sox system. Sulfate reduction was based on the presence of dsrAB^43^. Aerobic respiration was based on the presence of at least one set of genes encoding a cytochrome c oxidase. Nitrate/nitrite respiration was based on the presence of genes encoding one of several nitrate or nitrite reductases. Autotrophy was indicated by the presence of *acsAB* (indicating the Wood–Ljungdahl pathway), *cbbLS* (indicating the Calvin Cycle), or *nifJ*/*porCDAB/oorDABC* (indicating the reverse citric acid cycle). Further pathways and genes for chemotaxis, flagellar assembly, glycolysis, and aerobic methane or formate metabolism were also screened but not included in Fig. 3. All details of accession numbers screened for these genes and pathways are included in the header of Supplementary Dataset 1.

Relative genome bin quantities were obtained by mapping trimmed reads against a database of all binned contigs using BBMap, with coverage (average fold) for each contig taken from the program’s “covstats” output. A weighted mean was then calculated for the coverage of each genome bin, with the average fold value for each contig weighted based on the length of the contig.

To obtain a phylogenetic overview of the microorganisms sequenced in the metagenome, all trimmed reads were mapped against the SILVA small subunit rRNA database version 138.1^44^. These reads were then clustered and taxonomically classified by the SILVAngs pipeline version 1.4.6, also using SILVA version 138.1^44^).

### Cultivation and handling of *Acidovorax facilis* and *Nitrospina gracilis*

*Acidovorax facilis* DSM 649 was grown in nutrient broth (Merck) at 30 °C for approximately 1–2 weeks before use. A few drops of the *A. facilis* culture, pre-grown under oxic conditions and then flushed with N_2_ for 10 min, were added to glass slide chambers during assembly instead of the anoxic tap water. Slides were kept in moisture chambers at 16 °C for 12–24 h, so cable bacteria had time to reach from the sediment towards the edge of the slides. *A. facilis* cells were then analyzed by Raman microscopy as described below.

*Nitrospina gracilis* was cultivated on Red Sea salt-based marine mineral medium with vitamin supplements and 2 mM NO_2_^−^ at 28 °C in the dark without agitation^45^.

### Genome sequencing and analysis of *Acidovorax facilis*

DNA was extracted from *Acidovorax facilis* DSM 649 with the DNeasy PowerLyzer PowerSoil Kit (Qiagen), an Illumina library was prepared with the Nextera XT kit (Illumina), and the whole genome was sequenced on an Illumina MiSeq (see Lustermans et al.^46^ for details). Sequence quality was assessed using FastQC^36^ before trimming with Trimmomatic^37^ and assembly with SPAdes^47^. The genome was submitted to NCBI and is available under the accession number PRJNA849392.

The average nucleotide identity between the genome of DSM 649 and genome bin GS.4 was determined using the online ANI calculator (http://enve-omics.ce.gatech.edu/ani/) following the methodology of Goris et al.^48^.

### Raman microscopy

Spectra were recorded using a confocal LabRAM HR Evolution Raman microscope (HORIBA) equipped with a 500 mW, 532 nm neodymium-yttrium aluminum garnet laser, a 1064 nm laser for optical tweezing, an Andor EM CCD detector set to 500-650 nm emissions, grating 300 with a 2.8 cm^−1^ spectral resolution, pinhole set at 300 µm, and a 60× magnification water objective. Laser intensity was set to 25% maximum power for native flocking cells and to 10% for introduced *A. facilis* cells, with an acquisition time of 5–10 s per measurement. Native flocking cells with more accumulated material around were measured with higher laser intensities to acquire a higher signal-to-noise ratio. Cells of two morphologies, small rods (0.5–1 µm) and ovoid cells (0.5–1.5 µm), were captured at a 50 µm distance to a cable bacterium with bacterial flocking using the laser tweezer. Each captured cell was moved right next to the cable bacterium and measured, then moved to an area with no flocking cells, 50–100 µm away from the cable bacterium, and measured again to detect intensity differences in the 750 cm^−1^ band^18^ between the two measurements. Due to crowding around the cable bacterium, cells were often knocked out of the laser trap by other cells, and for this reason, only a single spectrum was captured at either position. All paired measurements that were incomplete or potentially compromised due to lost cells, trapping additional cells, or affected by cable bacteria movement, were removed. As the light had to be turned off during Raman measurements, leading to a short period where the trapped cell could be knocked out of the trap, paired spectra were kept only if they were similar within the pair, with no major shifts in the C–H region of the spectra (2800–3000 cm^−1^). Raman spectra of *A. facilis* cells were recorded for laser-trapped single, double, or clustered cells. To compare between measurements, the band intensity at 750 cm^−1^ was normalized by subtracting the median of the baseline from 735 to 740 cm^−1^ and from 760 to 765 cm^−1^.

The effect of laser trapping and moving cells on the Raman signal was investigated by using a pure culture of *Nitrospina gracilis*, under oxic and anoxic conditions. For the anoxic treatment, an aliquot of actively nitrifying *N. gracilis* was diluted 1:5 in fresh medium and transferred into Hungate tubes. The tubes were then flushed with N_2_ for 10 min, and a subsample was put into the same microscopy chamber as used in the previous experiments. Single cells were trapped with optical tweezers, measured, and moved 50–100 µm before measuring again to generate data pairs for each cell. Raman measurements were taken as described above with 10% laser intensity and an acquisition time of 10 s. Measurements were made in the center of the chamber to minimize oxygen contamination. The paired data were treated as described previously.

### Statistical analysis

Quantitative data from cell tracking, hybridization, and Raman microscopy were analyzed in R. For cell tracking and hybridization counts, Welch’s two-sample *t-*test was used. For Raman microscopy, a student’s *t*-test for dependent samples was used.

### Reporting summary

Further information on research design is available in the Nature Portfolio Reporting Summary linked to this article.

## Supplementary information

Supplementary Information

Description of Additional Supplementary Files

Supplementary Dataset 1

Supplementary Dataset 2

Supplementary Dataset 3

Supplementary Movie 1

Supplementary Movie 2

Reporting Summary

## Data Availability

Source data are provided in this paper. Microscopy data generated in this study have been deposited in the Zenodo database [10.5281/zenodo.7593818]. Metagenomic data (raw reads and the 27 metagenome-derived genomes) have been deposited in the NCBI database under accession number PRJNA730231. The genome of *Acidovorax facilis* DSM 649 has been deposited in the NCBI database under accession number PRJNA849392.

## Code availability

The R code used for the analysis of the microscopy data generated in this study has been deposited in the Zenodo database together with the microscopy data, as has the code to determine the pathway content of the metagenome-derived genomes.
