# Supplementary material for: Cable bacteria with electric connection to oxygen attract flocks of diverse bacteria
Source: Nat Commun. 2023 Mar 23;14:1614. doi: 10.1038/s41467-023-37272-8 (PMC10036481; doi:10.1038/s41467-023-37272-8)
Supplement: Supplementary file 1 — Supplementary Information [file 41467_2023_37272_MOESM1_ESM.pdf]

Supplementary information

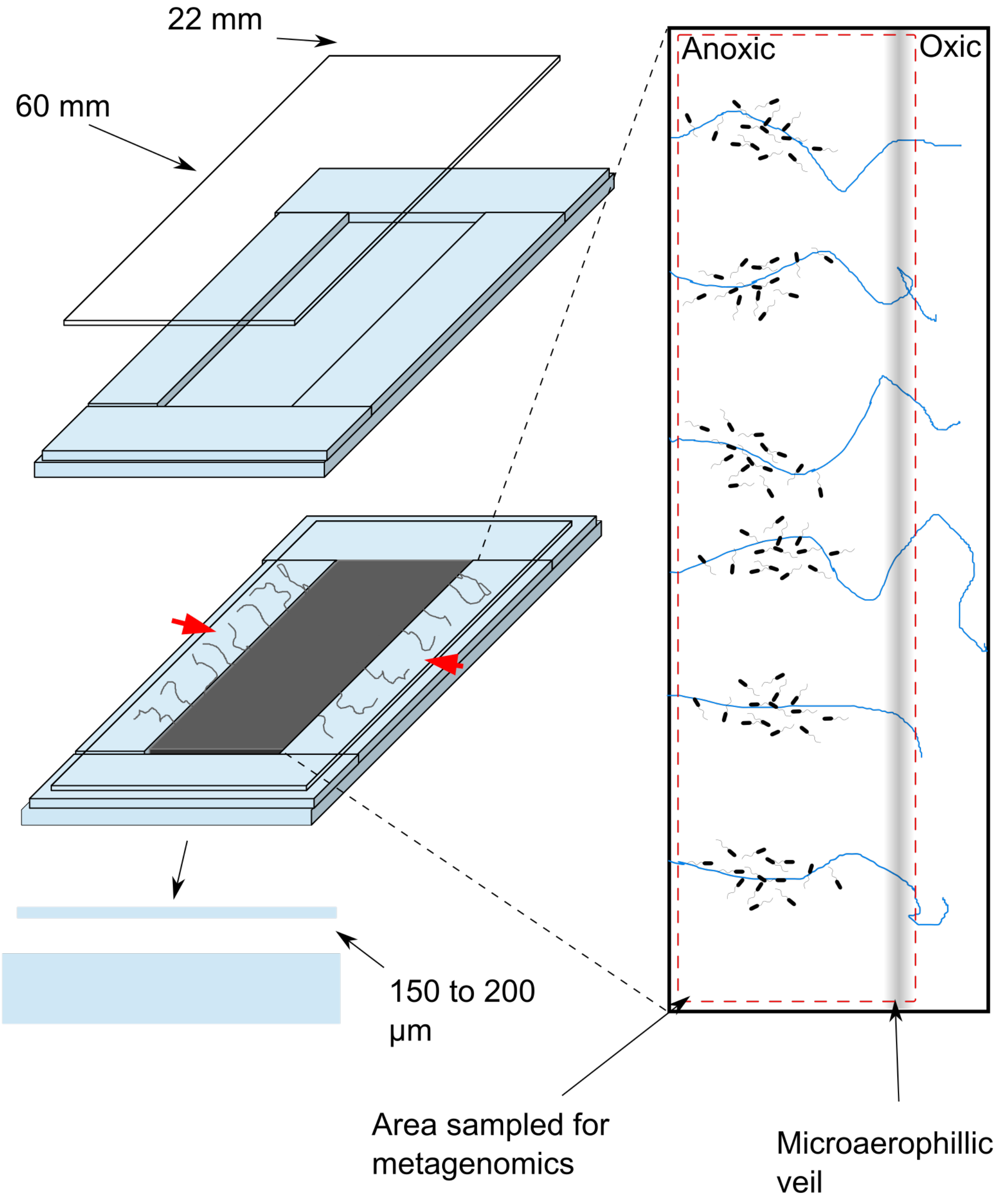

Figure S1 - Slide system used for discovery and investigation of the flocking bacteria around cable bacteria. Glass pieces are glued together to form a central trench. The trench is then filled with mud containing cable bacteria and covered with a long cover glass. Cable bacteria will then move out from the sediment in search of oxygen, spanning the glass surface, where their behavior and interactions with other bacteria can be observed. Insert shows the observation area, which is also the area sampled for metagenome sequencing. Modified from Supplementary Figure S1, Bjerg et al. 2016[1], with permission from the corresponding author.

Video	flocking cells	Cable bacterium length (in µm)	Cable bacterium cells	Flocking cells per cable bacterium cell
**Sample 1**	298	211	70.3	4.2
**sample 2**	51	290	58.0	0.9
**Sample 3**	193	203	40.6	4.8
**Sample 4**	172	266	53.2	3.2
**Sample 5**	79	129	25.8	3.1
**Sample 6**	83	131	26.2	3.2
**Sample 7**	46	132	26.4	1.7
**Sample 8**	111	286	57.2	1.9
**Sample 9**	77	130	26.0	3.0
**Sum**	1110	1778	383.7	2.9

Table S1 – Cell counts of flocking cells relative to cable bacteria cells. Data from 9 sample videos of both small and large type flocking cells, length of an average *Ca.* Electronema aureum GS cell is 5 µm. Cells were counted using cell tracking over 400 video frames for each video, and only tracks over 30 frames were counted. A full track counted as one cell. Tracks which never ventured within 20 µm of the cable bacteria filaments were not included. Videos were chosen for quality and lack of particles interfering with the counts. The cell counts are likely underestimating the true number of flocking cells.

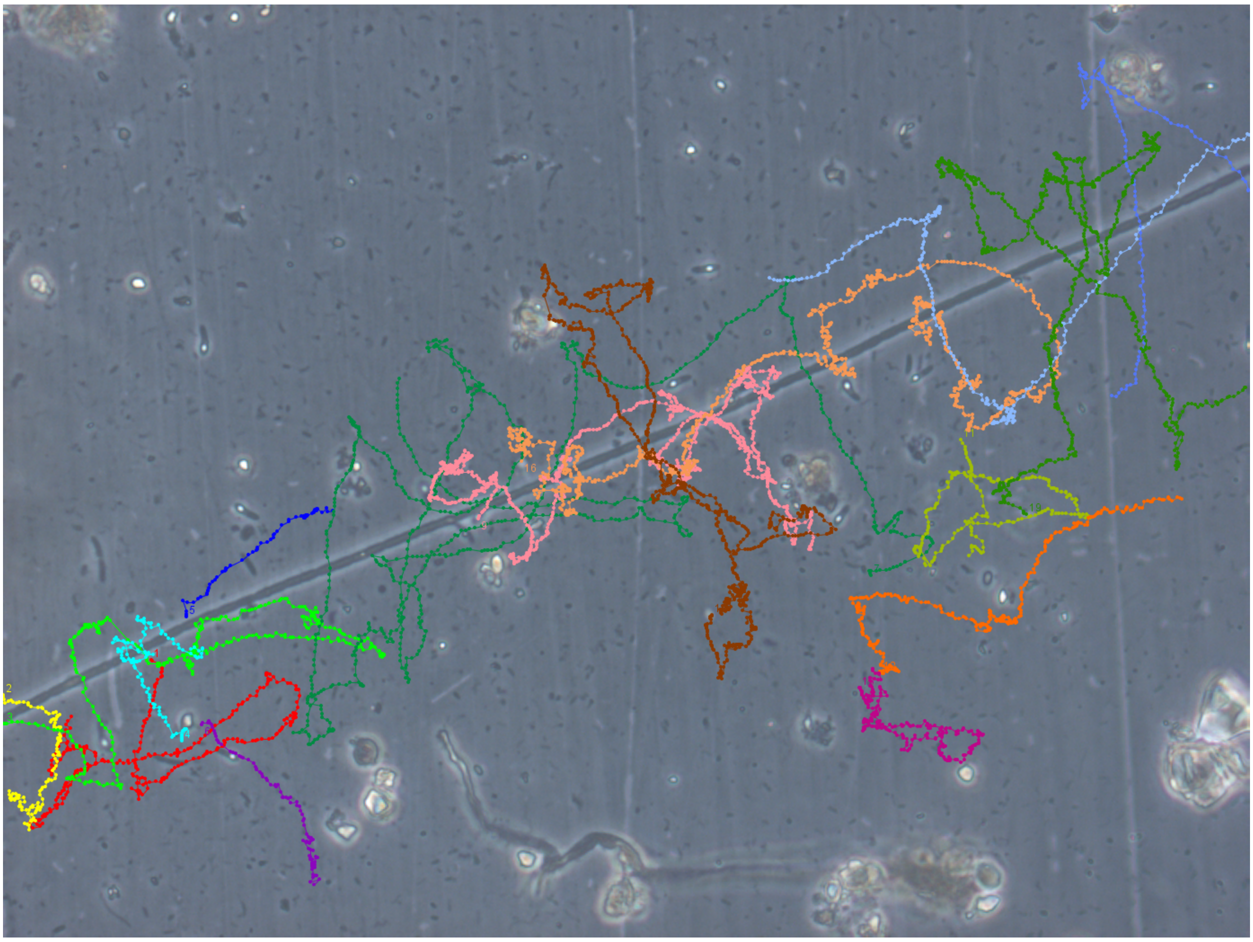

Figure S2 – Manual tracks from large type flocking bacteria. Each colour represents the trajectory of an individual cell swimming around the cable bacterium (diagonal, center) over the full length (400 seconds) of the movie. For automated tracks, Mtrack2 settings were adjusted until it would find the same tracks. Settings used were; *Size* "4-99 pixels", *minimum track* 253 *length* "50 frames", *maximum velocity* "20 pixels per frame". Source data are provided as a Source Data file.

**Close to cables <10 µm**				**Far from cable >10 µm**		
Fields of View	Cells hybridizing with EUBmix	Total cell count (DAPI)	Ratio of EUB/DAPI	Cells hybridizing with EUBmix	Total cell count (DAPI)	Ratio of EUB/DAPI
1	11	11	1.00	51	65	0.78
2	2	2	1.00	15	20	0.80
3	29	29	1.00	3	4	0.80
4	3	3	1.00	5	7	0.71
5	5	5	1.00	6	12	0.50
6	15	15	1.00	14	14	1.00
7	0	0	n/a	26	36	0.72

8	17	17	1.00	16	22	0.73
9	8	8	1.00	27	38	0.71
10	4	4	1.00	10	15	0.67
11	0	0	n/a	6	10	0.60
12	37	40	0.93	24	54	0.44
13	0	0	n/a	5	62	0.08
14	8	9	0.89			
15	1	1	1.00			
16	9	10	0.90			
17	9	11	0.82			
18	12	12	1.00			
19	18	21	0.86			
20	6	7	0.86			
21	10	11	0.91			
22	8	8	1.00			
23	4	10	0.40			
24	9	10	0.90			
25	10	14	0.71			
26	8	13	0.62			
27	17	20	0.85			
28	15	15	1.00			
29	6	9	0.67			
30	23	29	0.79			
31	15	15	1.00			
32	13	19	0.68			
33	17	18	0.94			
34	16	20	0.80			
35	13	27	0.48			
36	30	52	0.58			
37	29	44	0.66			
38	19	26	0.73			
39	22	23	0.96			
40	15	24	0.63			
41	23	36	0.64			
42	28	35	0.80			
43	19	22	0.86			
44	21	27	0.78			
45	28	36	0.78			
46	12	48	0.25			
St.dev	**9**	**13**		**13**	**20**	

Table S2 – Documentation of FISH counts. Difference in FISH detection frequency of cells close to or far from cable bacteria (N_{cells} = 1883, $N_{fields\ of\ view(close\ to)}$ = 46, $N_{fields\ of\ view(far\ from)}$ = 12, Welch Two Sample t-test; df = 17.052, p-value 0.01737)

Measured cable bacterium	Time for full dispersal (sec)
**Cut 1**	9
**Cut 2**	10
**Cut 3**	15
**Cut 4**	13
**Cut 5**	14
**Cut 6**	14
**Cut 7**	15
**Cut 8**	9
**Cut 9**	10
**Median for all cuts**	13
**Std**	2,4

Table S3 - Measurements of full dispersal time for small type flocking bacteria upon laser-cutting a cable bacterium. Full dispersal is defined as when no flocking bacteria were within 15 µm of the cable bacteria filaments. Only dispersal of small type flocking bacteria was measured (N = 9 cuts).

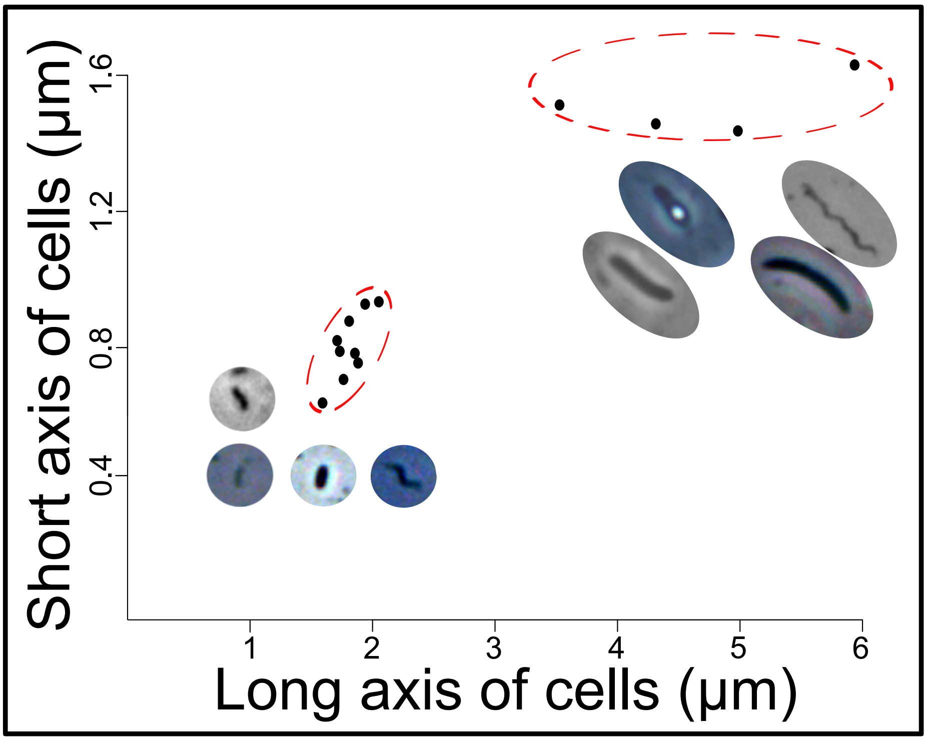

Figure S3 – Morphological diversity of flocking bacteria. Means of recorded sizes of flocking bacteria from the analysed videos, inserts show the diverse morphologies found ($N_{samples}$ = 11, $N_{flocking\ cells}$ = 2,712). Source data are provided in the Source Data file (data for Figure 1D).

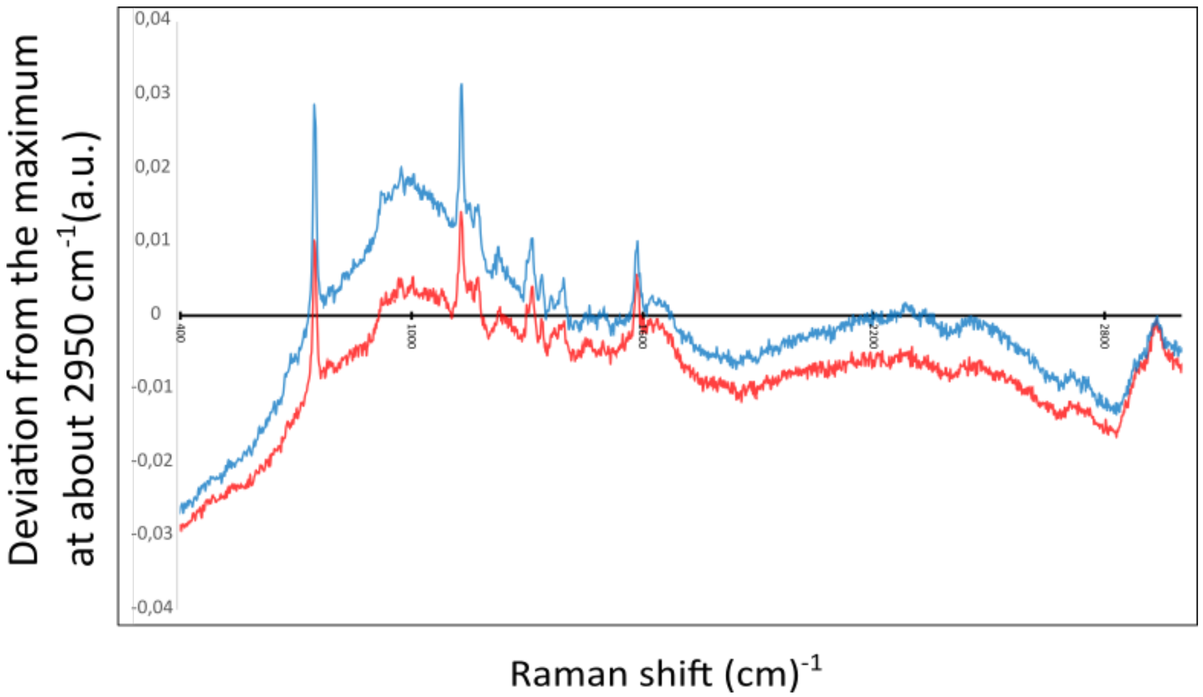

Figure S4 - Raman spectra of a flocking cell showing the characteristic spectra of C-type cytochrome hemes. Oxidized (red) and reduced (blue) states are represented as deviation from the maximum at about 2950 cm^{-1}. The maximum at 2950 cm^{-1} reflects C-H bonds, indicating biomass, which is constant between the two measurements, because the same cell is measured within a very short time period in two different distances from *Ca*. Electronema aureum GS. Source data are provided as a Source Data file.

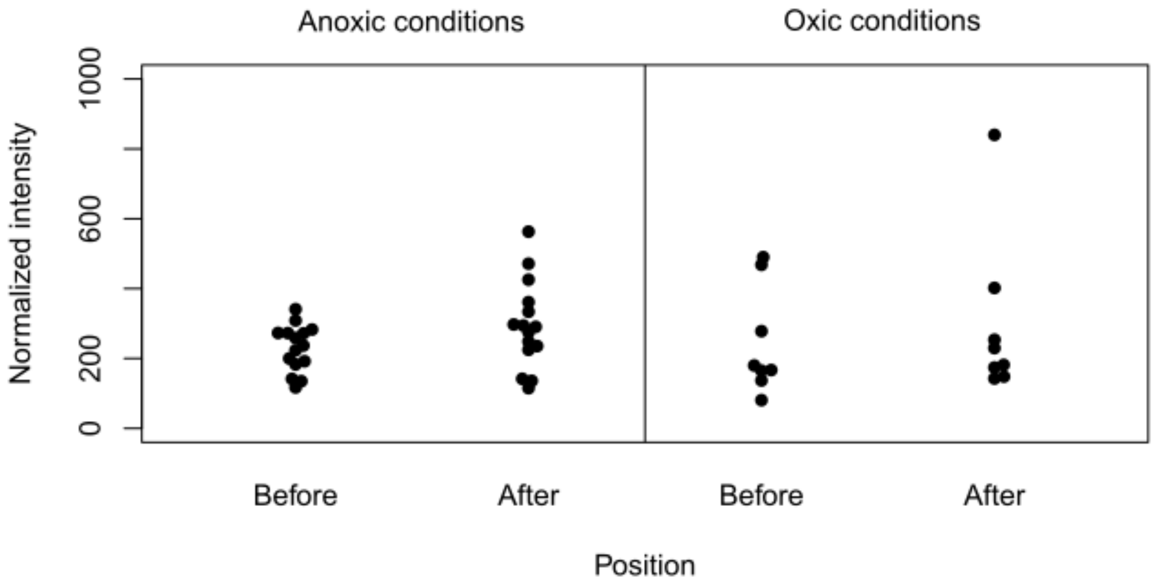

Figure S5 – Raman control experiments demonstrates stable Raman signals when moving cells by the laser tweezer. No significant change was observed in the normalized intensity (in arbitrary units) of the 750 cm^{-1} band of cytochrome c of *N. gracilis* cells when moved using the laser trap. ($N_{\text{anoxic cells}}$ = 14, p-value = 0.424; $N_{\textit{oxic cells}}$ = 8, p-value = 0.462, *t*-test for dependent samples). Source data are provided as a Source Data file.

		Estimates	
		Conservative	More realistic
Radius of filament *r*	cm	0.00007	0.00007
Radius of flock, *R*	cm	0.002	0.001
Exchange rate with filament, *P*	pmol cm^{-1} d^{-1}	140	7
Diffusion coefficient, *D*	cm^2 d^{-1}	0,4	1.6
Concentration difference, *ΔC*	nM	187	2
Turnover time, *t*	s	1.45	0.07

Table S4 - Radial diffusion around a cable bacterium filament. Estimations of concentration gradients and and turnover time for dissolved compounds exchanged between filament and flockers. Equations and assumptions are outlined in Supplementary Note 1.

Supplementary Note 1:

A compound that is exchanged by diffusion between a cable bacterium filament of radius *r* and a flock of radius *R* will form a concentration gradient that depends on the exchange rate of the compound per filament length, *P*, and the diffusion coefficient, *D*. According to Crank (1979)[3] this diffusion in a cylinder is described with the equation

$$P = 2\pi D(C_R - C_r)/\ln(R/r)$$

where C_R - C_r is the concentration difference, *ΔC*, between the surface of the filament and the flock. This rearranges into

$$\Delta C = P * \ln(R/r)/2\pi D$$

If the compound is completely depleted at one side, the turnover time, t, can be calculated from the radii and the diffusion coefficient[2]

$$t = R^2/2D * \ln(R/r)$$

From a recent report on the metabolism of individual cable bacterial from the same strain in a similar setting, a median electron generation of 140 pmol electrons per cm filament per day is calculated[3]. For a conservative estimate of how high *ΔC* may get, it is assumed that the entire electron flow is mediated by flocking cells placed 20 µm away from the filament (Figure 1B). Furthermore, a compound carrying only one electron equivalent and having a diffusion coefficient *D* of only 0.4 cm^2 d^{-1}, or about 4 times less than the diffusion coefficient for oxygen, is chosen. With these assumptions, the concentration difference *ΔC* amounts to no more than 187 nM and the turnover time *t* to 1.45 s (Table S4). This would be the highest concentration at the filament surface if the compound was completely depleted in the flock and the highest concentration at the flock in the opposite case with depletion at the filament surface.

In a supposedly more realistic scenario with cable bacteria mediating 90% of the electrons themselves, most flocking cells metabolizing closer to the filament, and the compound carrying 2 electron equivalents and diffusing as fast as oxygen, the concentration difference ΔC would be only 2 nM and turnover time t 0.07 s. In theory, many of the electron shuttles and other redox pairs occurring in sediments could thus serve as the mediating compound. For instance, excreted flavins, which have been found at concentrations beyond 1 nM in the top 5 cm of marine sediment[4].

Supplementary References:

1. Bjerg, J. T., Damgaard, L. R., Holm, S. A., Schramm, A. & Nielsen, L. P. Motility of electric cable bacteria. *Appl. Environ. Microbiol.* **82**, 3816–3821 (2016).
2. Crank, J. *The mathematics of diffusion*. Oxford University Press, 1979.
3. Scilipoti, S., Koren, K., Risgaard-petersen, N., Schramm, A. & Nielsen, L. P. Oxygen consumption of individual cable bacteria. *Sci. Adv.* **7**, 1–6 (2021).
4. Monteverde, D. R. et al. Distribution of extracellular flavins in a coastal marine basin and their relationship to redox gradients and microbial community members. Environ. Sci. Technol. 52, 12265–12274 (2018).
