# Supplementary material for: Cable bacteria with electric connection to oxygen attract flocks of diverse bacteria
Source: Nat Commun. 2023 Mar 23;14:1614. doi: 10.1038/s41467-023-37272-8 (PMC10036481; doi:10.1038/s41467-023-37272-8)
Supplement: Supplementary file 2 — Description of Additional Supplementary Files [file 41467_2023_37272_MOESM2_ESM.pdf]

Description of Additional Supplementary Files:

Movie S1 – Video captured of the flocking cells moving around a *Candidatus* Electronema aureum GS filament (center). Magnification is 400x, video is 200 frames, covering 17.6 seconds of flocking bacteria movement. For clarity, the video has been edited by subtracting the median value of each pixel from all frames, in effect removing non-moving parts of the video.

Movie S2 – Laser cut of *Ca.* Electronema aureum GS with active flocking. A cable bacterium filament reaching from sediment to oxygen was cut in the middle of the cable, in the middle of an active flock to illustrate the process. After the cut (frame 53), the left side of the cable is no longer connected to oxygen, while the right side is. Flocking cells disperse from the left side of the cable and are observed to migrate to the cable fragment on the right, that is still connected to oxygen connected. Magnification is 100x, video is 250 frames, covering 22 seconds.

Supplementary Dataset 1: Genome and literature search details.

Supplementary Dataset 2: Extracted 16S rRNA gene relative abundance from the metagenome.

Supplementary Dataset 3: Coverage of the genome bins.
