# Supplementary material for: Cable bacteria with electric connection to oxygen attract flocks of diverse bacteria
Source: Nat Commun. 2023 Mar 23;14:1614. doi: 10.1038/s41467-023-37272-8 (PMC10036481; doi:10.1038/s41467-023-37272-8)
Supplement: Supplementary file 8 — Reporting Summary [file 41467_2023_37272_MOESM8_ESM.pdf]

nature portfolio

Corresponding author(s): Jesper J. Bjerg, Andreas Schramm

Last updated by author(s): 2023-2-15

Reporting Summary

Nature Portfolio wishes to improve the reproducibility of the work that we publish. This form provides structure for consistency and transparency in reporting. For further information on Nature Portfolio policies, see our Editorial Policies and the Editorial Policy Checklist.

Statistics

For all statistical analyses, confirm that the following items are present in the figure legend, table legend, main text, or Methods section.

n/a	Confirmed	
☐	☒	The exact sample size (*n*) for each experimental group/condition, given as a discrete number and unit of measurement
☐	☒	A statement on whether measurements were taken from distinct samples or whether the same sample was measured repeatedly
☐	☒	The statistical test(s) used AND whether they are one- or two-sided<br>*Only common tests should be described solely by name; describe more complex techniques in the Methods section.*
☒	☐	A description of all covariates tested
☒	☐	A description of any assumptions or corrections, such as tests of normality and adjustment for multiple comparisons
☐	☒	A full description of the statistical parameters including central tendency (e.g. means) or other basic estimates (e.g. regression coefficient) AND variation (e.g. standard deviation) or associated estimates of uncertainty (e.g. confidence intervals)
☐	☒	For null hypothesis testing, the test statistic (e.g. *F*, *t*, *r*) with confidence intervals, effect sizes, degrees of freedom and *P* value noted<br>*Give P values as exact values whenever suitable.*
☒	☐	For Bayesian analysis, information on the choice of priors and Markov chain Monte Carlo settings
☒	☐	For hierarchical and complex designs, identification of the appropriate level for tests and full reporting of outcomes
☒	☐	Estimates of effect sizes (e.g. Cohen's *d*, Pearson's *r*), indicating how they were calculated

Our web collection on statistics for biologists contains articles on many of the points above.

Software and code

Policy information about availability of computer code

Data collection	ImageJ 1.53f51
Data analysis	R 4.1.2, MetaBat 0.25.4, BBMap 35.82, SAMtools 1.9, Trimmomatic 0.33, CheckM 1.1.3, GTDB-tk 1.5.1, IQTREE 1.6.12, kofamscan 1.2.0, BLAST 2.11.0+, silva ngs pipeline 1.4.6

For manuscripts utilizing custom algorithms or software that are central to the research but not yet described in published literature, software must be made available to editors and reviewers. We strongly encourage code deposition in a community repository (e.g. GitHub). See the Nature Portfolio guidelines for submitting code & software for further information.

Data

Policy information about availability of data

All manuscripts must include a data availability statement. This statement should provide the following information, where applicable:

- Accession codes, unique identifiers, or web links for publicly available datasets
- A description of any restrictions on data availability
- For clinical datasets or third party data, please ensure that the statement adheres to our policy

Source data are provided with this paper. Microscopy data generated in this study and the R code use for its analysis have been deposited in the Zenodo database [https://doi.org/10.5281/zenodo.7593818]. Metagenomic data have been deposited in the NCBI database under accession number PRJNA730231 [https://www.ncbi.nlm.nih.gov/bioproject/PRJNA730231]. Code to determine pathway content of the metagenome-derived genomes has also been deposited to the Zenodo record listed above. The genome of Acidovorax facilis DSM 649 has been deposited in the NCBI database under accession number PRJNA849392[https://

www.ncbi.nlm.nih.gov/bioproject/PRJNA849392].

Human research participants

Policy information about studies involving human research participants and Sex and Gender in Research.

Reporting on sex and gender	*Use the terms sex (biological attribute) and gender (shaped by social and cultural circumstances) carefully in order to avoid confusing both terms. Indicate if findings apply to only one sex or gender; describe whether sex and gender were considered in study design whether sex and/or gender was determined based on self-reporting or assigned and methods used. Provide in the source data disaggregated sex and gender data where this information has been collected, and consent has been obtained for sharing of individual-level data; provide overall numbers in this Reporting Summary. Please state if this information has not been collected. Report sex- and gender-based analyses where performed, justify reasons for lack of sex- and gender-based analysis.*
Population characteristics	*Describe the covariate-relevant population characteristics of the human research participants (e.g. age, genotypic information, past and current diagnosis and treatment categories). If you filled out the behavioural & social sciences study design questions and have nothing to add here, write "See above."*
Recruitment	*Describe how participants were recruited. Outline any potential self-selection bias or other biases that may be present and how these are likely to impact results.*
Ethics oversight	*Identify the organization(s) that approved the study protocol.*

Note that full information on the approval of the study protocol must also be provided in the manuscript.

Field-specific reporting

Please select the one below that is the best fit for your research. If you are not sure, read the appropriate sections before making your selection.

☐ Life sciences ☐ Behavioural & social sciences ☒ Ecological, evolutionary & environmental sciences

For a reference copy of the document with all sections, see nature.com/documents/nr-reporting-summary-flat.pdf

Ecological, evolutionary & environmental sciences study design

All studies must disclose on these points even when the disclosure is negative.

Study description	A laboratory study of sediment microbial communities containing cable bacteria involving laser cut and laser tweezer manipulations, and observations by light microscopy, Raman microscopy, and metagenomics.
Research sample	This research was performed on a laboratory enrichment of a sediment microbial community containing the cable bacterium Candidatus Electronema aureum. This enrichment culture was started in 2014 and has been maintained in Aarhus since. Interactions of cable bacteria with a bacterial isolate from a culture collection (Acidovorax facilis DSM 649) were also analyzed in this study.
Sampling strategy	No pre-determined sample size was used, as sampling was (a) serendipitous, based on occasional observations of a phenomenon and thus difficult to plan, and (b) based on an unexpected phenomenon that a single observation is sufficiently surprising to merit a discovery.
Data collection	Light microscopy data was collected by Jesper Bjerg using a digital camera connected to a microscope, Raman microscopy data was collected by Jesper Bjerg, Jamie Lustermans and Anna Mueller, metagenomic data was collected by technicians Britta Poulsen and Susanne Nielsen using an Illumina MiSeq sequencer.
Timing and spatial scale	This study was based on observations of a rare phenomenon - timing and spatial scale were dictated by how often the flocking was observed.
Data exclusions	Data were excluded if no flocking bacteria were observed, this was a pre-determined criterion. As described in the methods section of the paper, the majority of video data was excluded idue to quality insufficient for automated analysis. For paired Raman data, all pairs were kept unless it was obvious that there was a substitution of the cell.
Reproducibility	While the exact conditions leading to flocking were not identified in this study, flocking was observed a sufficient number of times (>1000) to show that the phenomenon is reproducible.
Randomization	This study was not suited to randomization as it was based on observations of a rare phenomenon - the fact that the phenomenon occurred at all was sufficient to drive the data collection and randomizing observations would not have changed conclusions in any way, but rather consumed vastly more time.
Blinding	*Describe the extent of blinding used during data acquisition and analysis. If blinding was not possible, describe why OR explain why blinding was not relevant to your study.*

Did the study involve field work? ☐ Yes ☒ No

Reporting for specific materials, systems and methods

We require information from authors about some types of materials, experimental systems and methods used in many studies. Here, indicate whether each material, system or method listed is relevant to your study. If you are not sure if a list item applies to your research, read the appropriate section before selecting a response.

Materials & experimental systems

n/a	Involved in the study
☒	☐ Antibodies
☒	☐ Eukaryotic cell lines
☒	☐ Palaeontology and archaeology
☒	☐ Animals and other organisms
☒	☐ Clinical data
☒	☐ Dual use research of concern

Methods

n/a	Involved in the study
☒	☐ ChIP-seq
☒	☐ Flow cytometry
☒	☐ MRI-based neuroimaging
